# Simple, Rapid *Mycobacterium ulcerans* Disease Diagnosis from Clinical Samples by Fluorescence of Mycolactone on Thin Layer Chromatography

**DOI:** 10.1371/journal.pntd.0004247

**Published:** 2015-11-19

**Authors:** Anita Wadagni, Michael Frimpong, Delphin Mavinga Phanzu, Anthony Ablordey, Emmanuel Kacou, Mirabelle Gbedevi, Estelle Marion, Yalan Xing, Vaddela Sudheer Babu, Richard Odame Phillips, Mark Wansbrough-Jones, Yoshito Kishi, Kingsley Asiedu

**Affiliations:** 1 Centre de Dépistage et de Traitement de l’Ulcère de Buruli d’Allada, Allada, Bénin; 2 Kumasi Centre for Collaborative Research in Tropical Medicine (KCCR), Kwame Nkrumah University of Science and Technology (KNUST), Kumasi, Ghana; 3 Institut Médical Evangélique de Kimpese, Kimpese, Democratic Republic of the Congo; 4 Department of Bacteriology, Noguchi Memorial Institute for Medical Research, University of Ghana, Accra, Ghana; 5 Centre de santé Saint michel, Zoukougbeu, Côte d’Ivoire; 6 Centre de Diagnostic et de Traitement de l'Ulcère de Buruli de Pobè, Fondation Raoul Follereau, Pobè, Bénin; 7 Department of Chemistry and Chemical Biology, Harvard University, Cambridge, Massachusetts, United States of America; 8 Department of Medicine, School of Medical Sciences, Kwame Nkrumah University of Science and Technology (KNUST), Kumasi, Ghana; 9 St George's University of London, London, United Kingdom; 10 Department of Control of Neglected Tropical Diseases, World Health Organization, Geneva, Switzerland; University of Tennessee, UNITED STATES

## Abstract

**Introduction:**

*Mycobacterium ulcerans* infection, known as Buruli ulcer, is a disease of the skin and subcutaneous tissues which is an important but neglected tropical disease with its major impact in rural parts of West and Central Africa where facilities for diagnosis and management are poorly developed. We evaluated fluorescent thin layer chromatography (f-TLC) for detection of mycolactone in the laboratory using samples from patients with Buruli ulcer and patients with similar lesions that gave a negative result on PCR for the IS*2404* repeat sequence of *M*. *ulcerans*

**Methodology/Principal findings:**

Mycolactone and DNA extracts from fine needle aspiration (FNA), swabs and biopsy specimen were used to determine the sensitivity and specificity of f-TLC when compared with PCR for the IS*2404*. For 71 IS*2404* PCR positive and 28 PCR negative samples the sensitivity was 73.2% and specificity of 85.7% for f-TLC. The sensitivity was similar for swabs (73%), FNAs (75%) and biopsies (70%).

**Conclusions:**

We have shown that mycolactone can be detected from *M*. *ulcerans* infected skin tissue by f-TLC technique. The technique is simple, easy to perform and read with minimal costs. In this study it was undertaken by a member of the group from each endemic country. It is a potentially implementable tool at the district level after evaluation in larger field studies.

## Author Summary

*Mycobacterium ulcerans* infection, known as Buruli ulcer, is a disease that affects the skin and underlying tissues. The organism responsible for the infection produces a potent toxin called mycolactone that causes extensive skin damage. Easy to perform and cheaper techniques are needed for diagnostic confirmation. We have evaluated fluorescent thin layer chromatography (fTLC) for detection of mycolactone in skin samples from patients with Buruli ulcer comparing them with samples from similar non-Buruli ulcer lesions that gave a negative result in the standard polymerase chain reaction (PCR) test for *M*. *ulcerans*. Fluorescent TLC had sensitivity of 73.2% and specificity of 85.7% when compared with PCR whether the skin sample was a swab, a biopsy or a fine needle aspirate. This study shows that mycolactone can be detected reliably from *M*. *ulcerans* infected skin tissue by the simple, low cost technique of fluorescent thin layer chromatography that could be developed for point of care use. It requires further evaluation in countries where Buruli ulcer disease is endemic.

## Introduction

*Mycobacterium ulcerans* infection, known as Buruli ulcer, is a disease of the skin and subcutaneous tissues which is an important but neglected tropical disease with its major impact in rural parts of West and Central Africa where facilities for diagnosis and management are poorly developed [1]. Since prevention is not possible in the absence of either an effective vaccine or a clear understanding of the mode of transmission, a major control strategy for Buruli ulcer is early detection and treatment, hinging on effective laboratory confirmation of suspected cases.

Standard routine laboratory techniques for the confirmation of Buruli ulcer disease are *M*. *ulcerans* isolation by culture, histopathology, smear microscopy for acid-fast bacilli (AFB) and polymerase chain reaction (PCR) for detection of the *M*. *ulcerans* specific insertion sequence IS*2404* [2]. Treatment decisions cannot be made on the basis of culture results because *M*. *ulcerans* grows too slowly (over 8–12 weeks) and histopathology is not available in most endemic countries. Microscopy for AFB can be done quickly at low cost but its sensitivity is only 40–60% so PCR for IS*2404* which has sensitivity of 92–95% has been established as the gold standard for case confirmation [2] [3] [4]. As PCR is sophisticated and expensive its use has been restricted to a small number of reference laboratories [5] and numerous studies have been carried out to develop a simple diagnostic test that can be used at point of care facilities [6] [7]. One such technique is fluorescent thin layer chromatography (f-TLC) [8] which targets mycolactone, a polyketide-like toxin produced by *M*.*ulcerans*. The toxin, which is not produced by any other human pathogen, is responsible for the characteristic necrosis in *M*.*ulcerans* infected lesions and it is present in the skin biopsies of mice and humans infected with *M*. *ulcerans* [9] [10] [11].

Using liquid chromatography-mass spectrometry (LC-MS) mycolactone has been detected in skin lesions in 77% of patients with untreated Buruli ulcer [11]. Mycolactone can be detected in human skin samples from patients with Buruli ulcer by conventional TLC as a band at retention factor value 0.23 but a similar, weaker band was seen in normal human skin [11]. Therefore the extraction procedure has been modified and a new method has been developed to detect mycolactone using 2-naphthylboronic acid to enhance the fluorescence of the molecule [9].

In the present study the new procedure has been evaluated for accuracy in samples from patients with Buruli ulcer and patients with similar lesions that gave a negative result on PCR for the IS2404 repeat sequence of *M*.*ulcerans*

## Materials and Methods

### Patients

**Fig 1:**
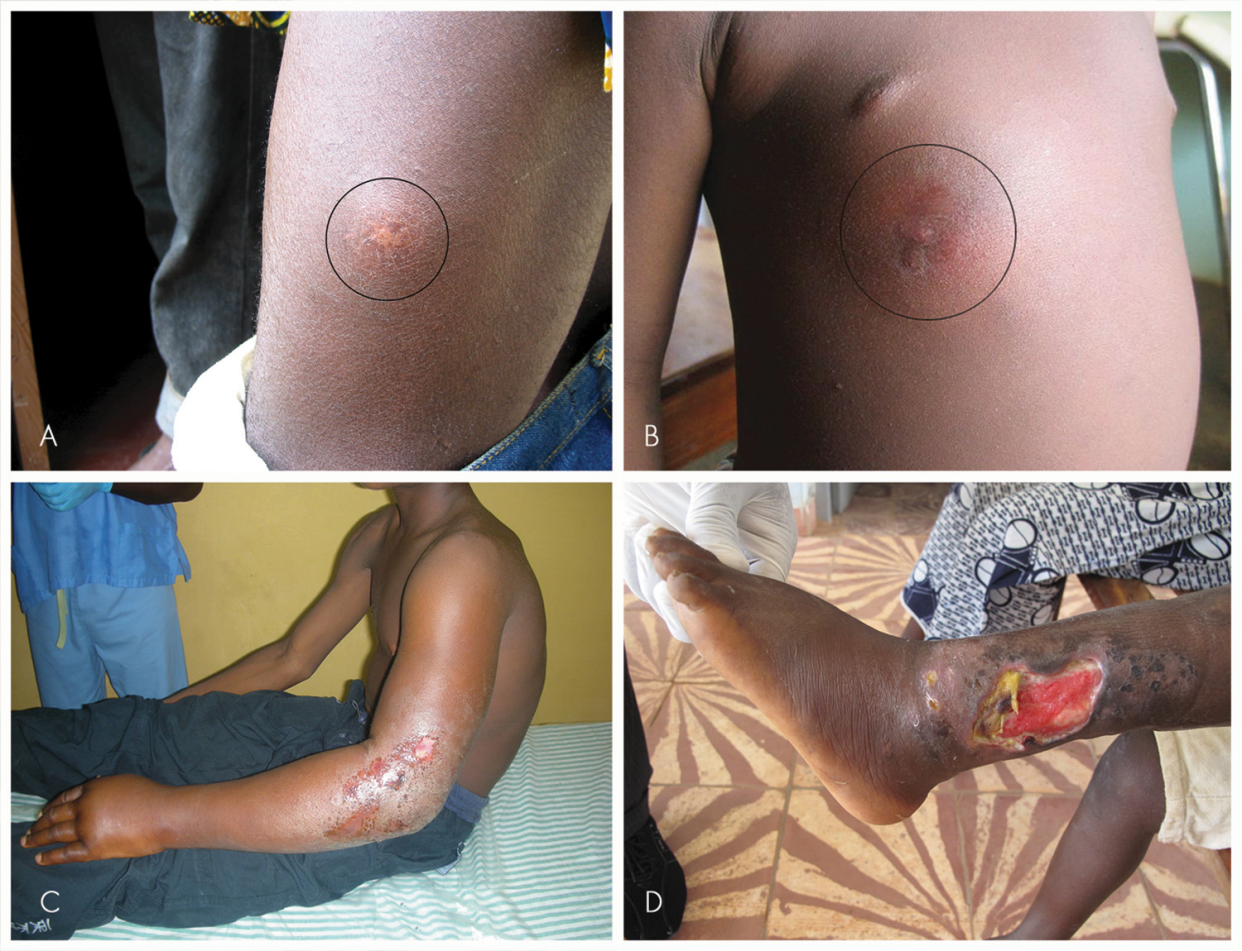
Clinical forms of Buruli ulcer showing a nodule (A), plaque (B), edema (C) and ulcer (D) (courtesy of World Health Organization).

Patients were recruited from Buruli ulcer treatment centres from January 2014 to June 2014 in Benin, DR Congo, Ghana and Côte d’Ivoire if they had a skin lesion suspected to be caused by *M*.*ulcerans* infection. Samples were collected by fine needle aspiration or swab according to whether the lesion was non-ulcerated or ulcerated respectively and by biopsy if obtained at surgery (Fig 1). If PCR for the *M*. *ulcerans* repeat sequence IS*2404* was positive they were included as Buruli ulcer disease patients. If the PCR was negative they were included in the control group.

### Sample preparation and shipment

Swabs, fine needle aspirates (FNA) or biopsy were put into O-ring seal plastic vials (Fisher Scientific) containing 1ml absolute ethanol. Vials were wrapped in aluminum foil and kept in the dark at room temperature. Samples from all the countries were transported to the Harvard laboratory within 3 weeks.

### Mycolactone extraction and fluorescent thin layer chromatography technique

Samples were processed by a modification of the published method [9] (S1 FTLC procedure and S2 FTLC procedure). Ethanol containing the dissolved sample was filtered through a cotton plug into a glass vial. The sample container was further rinsed with 1 mL ethyl acetate, which was added to the glass vial through a cotton plug, and the contents were evaporated to dryness under reduced pressure of about 10-15mmHg using a rotary evaporator (Rotavapor R-210, Buchi). To separate any contaminating solid from liquid, 100 μL hexane/ether (1:1) solution was added to the glass vial, rinsed and transferred by micro-syringe into a clean glass vial which was air-dried. After evaporation, 50 μL hexane/ether (1:1) was added to the dry sample and 15 μL of the resuspended sample was spotted onto a 3×6 cm fluorescent-dye free TLC plate (TLC Silica gel 60, EMD Millipore, Darmstadt, Germany; Gibbstown, NJ, USA) alongside 40 ng synthetic mycolactone A/B standard in ethyl acetate and a co-spot of 10 μL sample with 40 ng synthetic mycolactone A/B. The plate was developed in chloroform: hexane: methanol at a ratio of 5:4:1 until the leading edge reached the top of the plate, air-dried and dipped in 0.1 M 2-naphthylboronic acid solution in acetone, then heated for 60 seconds at 100°C on a hot plate. The glass side of the plate was wiped with acetone on a paper towel. The plate was placed on a UV lamp with a 365 nm filter. The fluorescent band at retention factor 0.23 from the patient sample was compared to that of the standards to confirm the presence of mycolactone. Two readers were made to confirm the mycolactone test result before test result reporting and were blinded to the PCR test result.

### Polymerase Chain Reaction

Duplicate samples were taken from each patient for PCR targeting the *M*.*ulcerans* IS*2404* repeat sequence. Samples were processed using the DNA extraction and PCR methods routinely used in diagnostic confirmation for patient care [12]. The amplification products were held at 4°C until they were processed further by agarose gel electrophoresis. The method of PCR included a negative extraction control and positive, negative and inhibition controls.

### Statistical analysis

GraphPad Prism 5 software was used for data analysis. Descriptive statistics were used to obtain general descriptive information such as the mean and ranges from the data. One sample analysis (Fisher’s exact test) was used to compare two proportions or groups. Contingency tables were used to calculate the sensitivity, specificity and the predictive values for the various laboratory techniques employed.

### Ethics statement

Ethical approval for the study was obtained from the School of Medical Sciences, Committee on Human Research, Publication and Ethics (CHRPE/AP/229/12). Written informed consent was obtained from the patient or their parent/guardian before samples were obtained.

## Results

Table 1 shows the characteristics of the patients and the type of sample taken from each. There were 71 IS2404 PCR positive samples and 28 PCR negative samples which were used as controls. The final diagnosis in the control samples is not known. The mean age of patients in the control group was 41 years compared with 13 years in the Buruli ulcer disease group.

**Table 1:** Characteristics of patients and sample type.

	PCR +ve	PCR-ve
	BU	Non BU
Number	71	28
Mean age (range)	13 (1–70)	41 (1–79)
Sex		
M	26	9
F	45	19
Sample type		
swab	49	15
FNA	12	3
biopsy	10	10

+ve—positive

-ve—negative

BU- Buruli ulcer

Out of 71 IS2404 PCR positive samples 52 were positive on fluorescent TLC giving a sensitivity of 73.2% (95% CI 61.4–83.1) (Table 2). 4 out of 28 true negatives gave a positive result on fTLC resulting in the specificity of 85.7% (95% CI 67.3–96.0). Thus 4/57 patients whose samples were positive by fTLC were false positives (7.0%). The positive predictive value (PPV) was 92.9% (95%CI 82.7–98.0) and the negative predictive value (NPV) 55.8% (95%CI 39.9–70.9). The sensitivity of fTLC was similar for swabs (73%), FNAs (75%) and biopsies (70%) (Table 3).

**Table 2:** Comparison of results of mycolactone detection by fluorescent TLC with PCR for *IS2404*.

	PCR +ve	PCR -ve	Total	Sensitivity %(95%CI)	Specificity %(95%CI)	PPV %(95%CI)	NPV %(95%CI)
**Mycolactone +ve**	52	4	**56**	**73.2**	**85.7**	**92.9**	**55.8**
**Mycolactone -ve**	19	24	**43**	(61.4–83.1)	(67.3–96.0)	(82.7–98.0)	(39.9–70.9)
**Total**	**71**	**28**					

+ve—positive

-ve—negative

PPV- positive predictive value

NPV- negative predictive value

**Table 3:** Sensitivity and specificity by sample type of fluorescent TLC for mycolactone compared to PCR for IS2404.

	PCR +ve	PCR -ve	Total	Sensitivity %(95%CI)	Specificity %(95%CI)
**Swab**					
mycolactone +ve	36	2	38	73.4 (58.9–85.1)	86.7 (59.5–99.4)
mycolactone -ve	13	13	26	73.4 (58.9–85.1)	86.7 (59.5–99.4)
**FNA**					
mycolactone +ve	9	0	9	75 (42.8–94.5)	100 (15.8–100)
mycolactone -ve	3	2	5	75 (42.8–94.5)	100 (15.8–100)
**Biopsy**					
mycolactone +ve	7	2	10	70 (34.8–93.3)	60 (40.0–97.2)
mycolactone -ve	3	3	6	70 (34.8–93.3)	60 (40.0–97.2)

+ve—positive

-ve—negative

Of the 4 false positives, 2 were on the lower limb in patients aged 42 and 62. The clinical diagnosis of these lesions is unknown and no culture of the underlying organism was performed. The other two were on the arms in a 12 year old male and in a 13 year old female.

## Discussion

We have shown that mycolactone can be detected in 73% of *M*.*ulcerans* infected samples by fluorescent thin layer chromatography. The technique was easy to perform and the result could be read within 1 hour. The sensitivity was higher than that of microscopy (30–60%) or culture (35–60%) and compared favourably with that of histology (82%)[13, 14]. There was no difference in sensitivity when FNA and swabs were compared although the number of FNA samples was small. In this study samples collected in endemic countries over a 3-week period were shipped to the laboratory at Harvard and stored before testing which may have impacted on the sensitivity of the assay. This can be investigated by field evaluation of the test in endemic countries.

The specificity was 87% but the implication of four false positives is that 4 patients would have received treatment for 8 weeks with daily injections of streptomycin and oral rifampicin with the known potential for side effects associated with those drugs. Two of these patients were more than 40 years old and had lesions on their lower limbs. Confirmatory PCR would usually be necessary in this group of patients because the differential diagnosis is broad. However the other two were children with lesions on upper limb which is more worrying. The correct diagnosis in these cases is not known and it remains possible that the PCR results were false negatives. A larger study in which false positives are followed up and diagnosed specifically is required to resolve this problem. The positive predictive value was 92.9% and the negative predictive value 55.8% but this needs to be interpreted with caution as samples from different populations were analysed and the population prevalence was not taken into account.

The extraction efficiency for mycolactone from human tissue is low at 10 to 20% (Caroline Demangel, personal communication) but fTLC is sensitive enough to detect less than 10ng mycolactone extracted from a mouse footpad [9]. The number of FNA samples was low in the present study but mycolactone was detected in these in the same proportion as from swabs and biopsies. This is an important consideration since more non-ulcerated early lesions are seen when campaigns to increase awareness of Buruli ulcer are successful.

Development of a diagnostic test for *M*.*ulcerans* disease that can be carried out in local treatment centres is a high priority and at a consultative meeting organized by World Health Organisation/Neglected Tropical Diseases (WHO/NTD) and Foundation for Innovative New Diagnostics (FIND) in Geneva November 2013 [15], fTLC was identified as a promising technique with potential for implementation at the district level. Further studies are planned with the support of FIND and WHO (NTD and TDR) to address the logistics of introducing this test in endemic countries, to confirm its sensitivity and to investigate its specificity.

## Supporting Information

S1 FTLC procedure Schematic Diagram of F-TLC Analysis of Swab or FNA Samples. (PDF)

S2 FTLC procedure Schematic Diagram of F-TLC Analysis of a Biopsy Samples. (PDF)

S1 Checklist STARD checklist for reporting of studies of diagnostic accuracy. (DOC)
