# Supplementary material for: Simple, Rapid Mycobacterium ulcerans Disease Diagnosis from Clinical Samples by Fluorescence of Mycolactone on Thin Layer Chromatography
Source: PLoS Negl Trop Dis. 2015 Nov 19;9(11):e0004247. doi: 10.1371/journal.pntd.0004247 (PMC4652903; doi:10.1371/journal.pntd.0004247)
Supplement: S2 FTLC procedure — (PDF) [file pntd.0004247.s002.pdf]

Schematic Diagram of F-TLC Analysis of Biopsy Samples

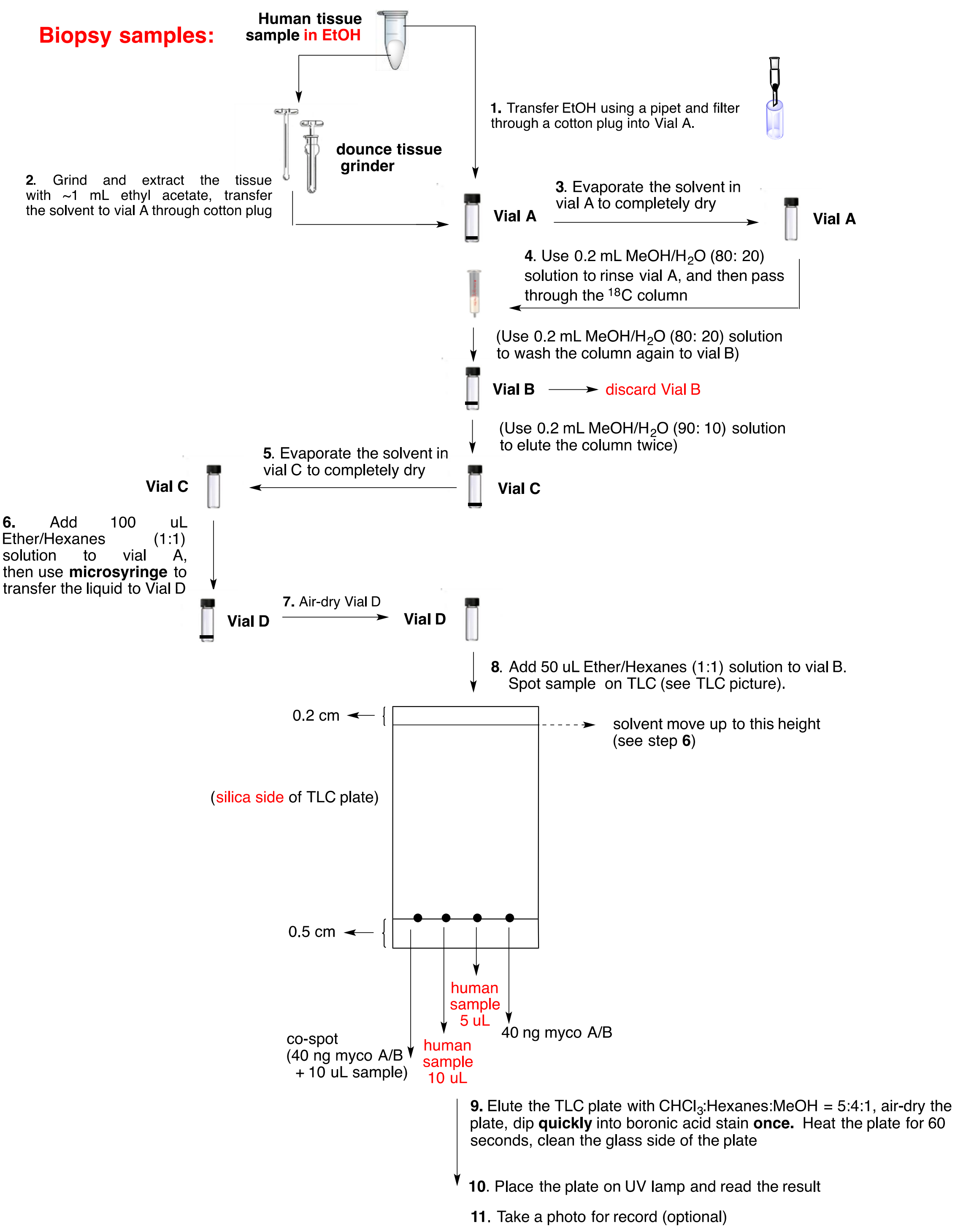

Procedure of F-TLC Analysis of Biopsy Samples

1. Transfer EtOH to a glass vial: Transfer EtOH (list #1) solution using a pipet (list #3) and filter through a cotton plug[1] (list #4) into a glass vial A (list #2a). Evaporate the solvent under reduced pressure (rotatory evaporator, list #17).

2. Grind and extract the tissue: Take out the tissue using a forceps to a dounce tissue grinder (list #10), add 1 mL EtOAc (list #1) to the grinder, grind about 15 times, then use a glass pipet to transfer the EtOAc solution and filter the EtOAc solution through a cotton plug into vial A.

3. Evaporate the solvent in vial A under reduced pressure to completely dry.

4. Pass through the ^{18}C column: a). Add 0.2 mL MeOH/H_2O (80:20) solution to vial A, then transfer the solution with a glass pipet to a ^{18}C column (see list #6), elute the column with 0.2 mL MeOH/H_2O (80:20) solution to vial B, and discard the eluent in vial B. b) Elute the column again with 0.2 mL MeOH/H_2O (90:10) solution **twice**, collect these eluents into glass vial C.

5. Evaporate completely the solvent in vial C under reduced pressure (rotatory evaporator followed by blowing Nitrogen gas).[2]

6. Separate the solid and liquid: Add 0.1 mL (1:1)-hexanes/ether solution to vial C, rinse the vial, use micro-syringe (see list #11) to transfer only the liquid[3] to Vial D,

7. Air-dry the D completely.

8. Spot sample on TLC: On a TLC plate (see list #5),[4] draw a line using a pencil 0.5 cm above the bottom of the plate. Mark four spots that have equal distance between each other (see picture). Add 50 uL (1:1) hexanes/ether (list #1) to Vial C, use capillary pipet (see list #7) to take 10 uL and 5 uL of the solution to spot human sample on TLC plate. Try to make the sample spot as small as possible[5] on the TLC plate, also spot 40 ng (4

---

[1] Cotton plug is made by a small cotton bar and a 5 3/4″ disposable glass pipet. It is also used in step 2.

[2] Make sure the solvent is completely removed; you could see white or light yellow solid materials formed on the wall of the glass vial.

[3] The reason for using micro-syringe is to avoid transfer the solid which is responsible for the fluorescent background impurities. Make sure to only transfer the liquid to vial C, leave the solid on the wall of the vial.

[4] Cut the TLC plate to size of 3 cm x 6 cm, see list #18 for TLC cutter.

[5] Blow air to dry the solvent quickly after each spot.

uL)- standard mycolactone A/B solution.[6] For co-spot, spot 10 uL human sample plus 40 ng (4 uL)- standard mycolactone A/B solution at the same spot on TLC plate.

9. Elute the TLC plate: Elute the TLC plate using developing chamber (list #14) with $CHCl_3$:Hexanes:MeOH = 5:4:1 (list #1) solution[7] (solvent front to 0.2 cm below the top of the TLC plate), then air-dry the plate, quickly dip the plate into boronic acid stain[8] once, then heat the plate at 100 °C using heating plate (list #15) for 60 seconds, clean the backside (glass) of the TLC plate[9]

10. Place the TLC plate on UV light (list #9), compare the intensity of the florescent spot and read the results.

11. Take a picture (optional): Take a picture in dark (Cannon EOS, see list #12, M mode, ISO 100, 4 second). Subject the picture for Photoshop (list # 13) enhancement.

---

[6] Standard mycolactone A/B solution for TLC is 10 ng/uL, made from the ampule of 0.1 mg/mL synthetic mycolactone A/B sample. Take 0.1 mL of the ampule solution to another vial (list #2b), add 0.9 mL EtOAc to give 10 ng/uL mycolactone A/B standard solution.

[7] Make this elution solution fresh every day before use.

[8] 0.13 M of the 2-naphthylboronic acid solution in acetone: 2.2 gram of the 2-naphthylboronic acid dissolved in 100 mL acetone, keep in a glass jar (list #16).

[9] Use paper towel and acetone to wipe the glass surface.
